# Supplementary material for: Detection of Recombinant Hare Myxoma Virus in Wild Rabbits (Oryctolagus cuniculus algirus)
Source: Viruses. 2020 Oct 5;12(10):1127. doi: 10.3390/v12101127 (PMC7600370; doi:10.3390/v12101127)
Supplement: Supplementary file 1 [file viruses-12-01127-s001.pdf]

Detection of Recombinant Hare Myxoma Virus in Wild Rabbits (*Oryctolagus cuniculus algirus*)

Fábio A. Abade dos Santos [1,2,3,*], Carina L. Carvalho [1], Andreia Pinto [4], Ranjit Rai [4], Madalena Monteiro [1], Paulo Carvalho [1], Paula Mendonça [1], Maria C. Peleteiro [2], Francisco Parra[3], Margarida D. Duarte [1,2]

[1] Instituto Nacional de Investigação Agrária e Veterinária, Av. da República, Quinta do Marquês, 2780-157 Oeiras, Portugal; carina.carvalho@iniav.pt (C.L.C.); madalena.monteiro@iniav.pt (M.M.); paulo.carvalho@iniav.pt (P.C.); paula.mendonca@iniav.pt (P.M.); margarida.duarte@iniav.pt (M.D.D.)

[2] CIISA, Faculdade de Medicina Veterinária, Universidade de Lisboa, Avenida da Universidade Técnica, 1300-477 Lisboa, Portugal; mcpelet@fmv.ulisboa.pt (C.P.)

[3] Instituto Universitario de Biotecnología de Asturias (IUBA), Departamento de Bioquímica y Biología Molecular, Universidad de Oviedo, 33006 Oviedo, Spain.; fparra@uniovi.es

[4] Paediatric Respiratory Medicine, Primary Ciliary Dyskinesia Centre, Royal Brompton & Harefield NHS Trust, London SW3 6NP, United Kingdom.; a.pinto@rbht.nhs.uk (A.P.); r.rai@rbht.nhs.uk (R.R.)

* Correspondence: fabio.abade@iniav.pt; Tel.: +351-21-440-3500

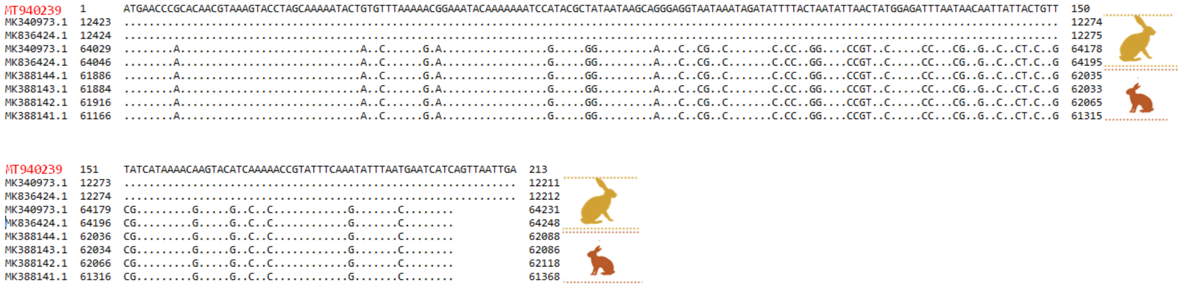

Figure S1. Nucleotide alignment between the M066L from sequence MT940239 with the homologous sequences of M006L from other hare recombinant strains (MK836424 and MK340973) (first to third sequences) and with the M066R from hare and classic Myxoma virus (MYXV) strains from rabbit (fourth to ninth sequences). The hare yellow icon delimits the sequences from strains isolated from hares, and the rabbit orange icon delimits the rabbit strains. The nucleotide position relative to the query (MT940239) indicates the insertion location (12423nt or 12424nt), and the location of M066R sequence (611884nt to 64046nt) in the complete genome.

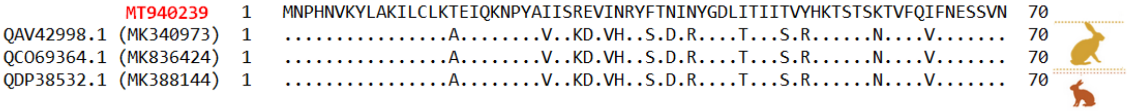

Figure S2. Alignment of the truncated M066L protein sequence from a natural recombinant MYXV (deduced from sequence MT940239) with the homologous regions of M066R proteins deduced from hare recombinant MYXV strains (MK836424 and MK340973) and classic MYXV strain (MK388144).
